# Lateral Flow Assay for Preeclampsia Screening Using DNA Hairpins and Surface-Enhanced Raman-Active Nanoprobes Targeting hsa-miR-17-5p

**DOI:** 10.3390/bios14110535

**Published:** 2024-11-05

**Authors:** Ka Wai Ng, Siddhant Jaitpal, Ngoc Nhu Vu, Angela Michelle T. San Juan, Sayantan Tripathy, Rohit Sai Kodam, Abhishek Bastiray, Jae-Hyun Cho, Mahua Choudhury, Gerard L. Coté, Samuel Mabbott

**Affiliations:** 1 Department of Biomedical Engineering, Texas A&M University, 5045 Emerging Technologies Building, College Station, TX 77843, USA; ngkerry1997@tamu.edu (K.W.N.); sjaitpal@tamu.edu (S.J.); nvu1@exchange.tamu.edu (N.N.V.); angelasanjuan08@tamu.edu (A.M.T.S.J.); tripathysayantan@gmail.com (S.T.); rohitsaikodam@tamu.edu (R.S.K.); gcote@tamu.edu (G.L.C.); 2 Department of Biochemistry and Biophysics, Texas A&M University, College Station, TX 77843, USA; bastiray@exchange.tamu.edu (A.B.); jaehyun.cho@ag.tamu.edu (J.-H.C.); 3 Irma Lerma Rangel College of Pharmacy, Texas A&M University, 159 Reynolds Medical Building, College Station, TX 77843, USA; mchoudhury@tamu.edu; 4 Center for Remote Health Technologies & Systems, Texas A&M Engineering Experimentation Station, 1041 Emerging Technologies Building, College Station, TX 77843, USA; 5 Department of Electrical Engineering, Texas A&M University, College Station, TX 77843, USA

**Keywords:** microRNAs, preeclampsia, DNA hairpin, surface-enhanced Raman scattering, lateral flow assay, nanocomposites, paper microfluidics

## Abstract

Preeclampsia (PE) is a serious complication that poses risks to both mothers and their children. This condition is typically asymptomatic until the second or even third trimester, which can lead to poor outcomes and can be costly. Detection is particularly challenging in low- and middle-income countries, where a lack of centralized testing facilities coincides with high rates of PE-related maternal mortality. Variations in the levels of hsa-miR-17-5p have been identified as constituting a potential early indicator for distinguishing between individuals with PE and those without PE during the first trimester. Thus, developing a screening test to measure hsa-miR-17-5p levels would not only facilitate rapid detection in the early stages of pregnancy but also help democratize testing globally. Here, we present a proof-of-principle lateral-flow assay (LFA) designed to measure hsa-miR-17-5p levels using DNA-hairpin recognition elements for enhanced specificity and nanoprobes for sensitive surface-enhanced resonance Raman scattering (SERS) signal transduction. The theoretical limit of detection for hsa-miR-17-5p was 3.84 × 10^−4^ pg/µL using SERS.

## 1. Introduction

PE is a progressive hypertensive multisystem disorder that occurs during pregnancy and is linked to abnormal placentation [1]. This condition involves atypical formation of uterine arteries, which can lead to endothelial dysfunction, oxidative stress, and systemic inflammation [2]. PE affects 2–8% of pregnancies worldwide and is a major contributor to maternal and perinatal mortality and morbidity [3], with 76,000 maternal and over 500,000 fetal deaths each year [4]. In the U.S., PE affects about 4.6% of all pregnancies [5] and is a leading cause of premature birth and contributor to low birth rates, adding USD 2.18 billion annually to healthcare costs [6].

Global definitions of PE vary among organizations. The International Society for the Study of Hypertension in Pregnancy (ISSHP) defines PE as gestational hypertension and proteinuria after 20 weeks, including organ dysfunctions like neurological issues, pulmonary edema, acute kidney injury, and liver problems [4]. The National Institute for Health and Care Excellence (NICE) [5] includes new-onset hypertension after 20 weeks, proteinuria, and other organ dysfunctions such as renal insufficiency and uteroplacental dysfunction in its criteria. In contrast, the American College of Obstetricians and Gynecologists (ACOG) [1] has updated its guidelines such that proteinuria is no longer required for a PE diagnosis, focusing instead on elevated blood pressure paired with complications like thrombocytopenia, renal insufficiency, impaired liver function, pulmonary edema, or neurological/visual symptoms.

Modern definitions of PE now include organ dysfunction, HELLP syndrome, and uteroplacental dysfunction, beyond just high blood pressure and proteinuria [1]. This expanded understanding facilitates more comprehensive diagnosis and management, potentially improving outcomes [7].

Advances in medical imaging, such as uterine arterial Doppler ultrasound and placental function MRI, enhance diagnosis by assessing reduced oxygenation levels in the placentas of women with preeclampsia [8]. Further, the discovery of biomarkers like sFLT-1 and PlGF [9] are providing insights into the underlying mechanisms and enabling early detection and accurate risk categorization [10]. Despite these advancements, challenges such as symptom variability, asymptomatic cases, and inconsistent diagnostic criteria complicate PE screening and monitoring. In regions with limited prenatal care, these challenges worsen, emphasizing the need for research into biomarkers and multi-omics to improve understanding, treatment, and Point-of-Care (PoC) monitoring of PE [11].

Research indicates that miRNAs, particularly hsa-miR-17-5p, are promising epigenetic biomarkers for the early detection of PE and other vascular disorders [12,13]. These miRNAs regulate gene expression and are circulating, meaning they can be detected in blood as well as other biological media. hsa-miR-17-5p is a critical component of the hsa-miR-17-92 cluster whose increased expression is linked to reduced aromatase protein expression and inhibited trophoblast differentiation, negatively affecting placental vascularization [12]. A study has shown that differential expression of miRNAs, including a significant 1.5-fold change in eight specific miRNAs, can distinguish PE from normal cases [14]. Notably, a 3-fold increase in hsa-miR-17-5p expression was observed in first-trimester PE patients compared to those with normal pregnancies [15], underscoring the diagnostic potential of miRNAs in PE screening.

Building on the diagnostic potential of miRNAs for conditions like PE, our research aims to harness these biomarkers within a screening test framework. Human serum, as opposed to other bodily fluids like urine or saliva, offers a richer source of miRNAs [16]. Despite low levels and preservation challenges, miRNAs like hsa-miR-17-5p are key gene regulators and sensitive disease biomarkers, offering early detection before conventional protein biomarkers appear.

To enable the early detection of PE by targeting hsa-miR-17-5p, we present a lateral-flow assay approach [17]. Our system comprises DNA hairpin detection and capture sequences designed to trigger the formation of a sandwich assay in the presence of hsa-miR-17-5p. We use silica-coated gold nanostar nanoprobes for colorimetric and surface-enhanced Raman scattering (SERS) signal transduction. Harnessing the signal enhancement effects of SERS helps alleviate sensitivity issues associated with detecting miRNA in low quantities [18,19]. Therefore, SERS is a signal amplification method that has been successfully used with LFA systems to achieve sensitive detection in areas like infectious diseases [20] and food safety [21].

## 2. Materials and Method

### 2.1. Polyacrylamide Gel Electrophoresis (PAGE)

Native PAGE was used to examine the hybridization of sequences to the miRNA target. To prepare a 15% polyacrylamide gel, we first mixed an acrylamide/bis-acrylamide solution (29:1, BioRad (Hercule, CA, USA), 1610158) with an equal volume of 2× Tris/Borate/EDTA (TBE) buffer. Next, we added 10% (*w*/*v*) ammonium persulfate (APS) and 100% (*v*/*v*) TEMED (*N*,*N*,*N*′,*N*′-tetramethylethylenediamine) at ratios of 100:1 and 1000:1, respectively. All hairpin sequences and miRNAs evaluated had a final concentration of 1 µM. To form the hairpins, hairpin oligonucleotides were heated to 95 °C for 5 min and then left to cool to room temperature (RTP). Hybridization reactions between the hairpins and miRNA were carried out in 1× SSPE buffer containing 0.15 M NaCl, 0.01 M NaH_2_PO_4_, and 1 mM EDTA at pH 7.4 and left to proceed for 2 h at RTP. Glycerol was added to each reaction to bring the total volume to 12 µL before being loaded into the wells of the gel.

### 2.2. Synthesis of SERS-Active Silica-Coated Gold Nanostar (SiO_2_-AuNS) Nanoprobes

#### 2.2.1. Gold Nanostar (AuNS) Synthesis

Gold nanoparticles (AuNPs) with a diameter of 13–16 nm were synthesized according to the Turkevich method [9], whereby 100 mL of 0.1 mM aqueous auric acid solution was prepared and heated to boiling. While maintaining vigorous stirring, an aqueous trisodium citrate solution (0.85 mL, 10 mg/mL) was added to boiling auric acid solution. The reaction mixture was boiled for 15 min, causing the solution to change color from light yellow to red, indicating the formation of AuNPs. The particles were left to cool to RTP before being transferred to a glass bottle for storage at 4 °C. To prepare the 80 nm AuNS, 1 mL of AuNPs (OD~0.7) was transferred to 50 mL of 0.25 mM auric acid solution followed by 50 μL of 1 M HCl. After, 2 mL of 0.5 mM AgNO_3_ and 2 mL of 25 mM of ascorbic acid were added simultaneously and immediately vortexed for 30 s. A color change from a light red to blue signified the successful synthesis of the AuNS. The AuNS solution was transferred into a 50 mL tube and centrifuged for 15 min at 4000 rpm. The supernatant was removed, and the pellet was resuspended in DI water. The nanoparticles were then filtered through a 0.22 μm nitrocellulose membrane.

#### 2.2.2. Raman Reporter Conjugation and Silica Shell Formation

The method of silica coating was adapted from Yuan, H., et al. [22]. In brief, 800 mL of reaction buffer was prepared by mixing 720 mL of isopropanol (IPA), 9.6 mL of tetraethyl orthosilicate (TEOS), 200 μL of 1 M 4-mercaptobenzoic acid (4-MBA) solution, and 72 mL of deionized (DI) water and stirred for 30 min at 500 rpm. 4-MBA was purposely selected as the Raman reporter since its vibrational peaks did not overlap with the Raman signal of nitrocellulose present in the commercially sourced LF strips. Subsequently, 3.6 mL of AuNS (OD~0.4) was resuspended in 36 mL of ethanol (EtOH) containing 3 mL of aqueous ammonia hydroxide and mixed for 5 min at 500 rpm. The AuNS particles were then mixed with 105.6 mL of reaction buffer for 20 min. Afterward, the solution was distributed into 3 individual 50 mL tubes and centrifuged at 12,000 rpm for 20 min to facilitate the formation of dark-blue pellets. The supernatant was removed before the SERS-active SiO_2_-AuNS nanoprobes were suspended in 1 mL of EtOH. The mixture was then transferred to 2 mL microcentrifuge tubes for washing, which was repeated four times in ethanol and two times with DI water. Eventually, the nanoprobes were resuspended in nuclease-free water.

#### 2.2.3. DNA and Streptavidin Functionalization of the SiO_2_-AuNS Nanoprobes

The protocol used for conjugation of streptavidin to the surface of the nanoprobes was adapted from Schiestel, T. et al. [23]. A total of 500 μL of nanoprobes (OD~0.4) was first mixed with 10% ammonium hydroxide, and then 1.5 μL of 34% 3-aminopropyltriethoxysilane (APTES) was added. The conjugation mixture was then placed on a shaker for 3 h at RTP. The particles were then centrifuged twice at 4000 rpm for 10 min, and, each time, the supernatant was removed and replaced with DI water. After washing, 10 μL of 10 mM *N*-ethyl-*N*′-(3-(dimethylamino) propyl) carbodiimide, 10 μL of 25 mM N-hydroxysuccinimide, and 5 μL of 1 mg/mL streptavidin (Pierce™ Streptavidin catalog no.: 21145) were added to the nanoprobe suspensions and incubated overnight at 4 °C. The streptavidin-conjugated nanoprobes were then washed according to the previous protocol; however, after the final wash, the particles were resuspended in 500 μL of 1× phosphate saline buffer (PBS).

Conjugation of the biotinylated DNA sequences to the streptavidin-functionalized nanoprobes was carried out as follows: 10 μL of 100 μM biotinylated DNA hairpin (synthesized by Integrated DNA Technologies (Coralville, IA, USA)) was incubated with 490 μL of nanoprobes (OD~0.4, eluted in 1× PBS, 0.05% Tween^®^20) at RTP for 1 h and then cleaned using an Amicon^®^ ultra centrifuge filter (100 kDa cut-off) to remove excess hairpins. DNA-conjugated SiO_2_-AuNS particles (DNA-SiO_2_-AuNS) were then eluted in 1× Tris-EDTA buffer with, 0.05 % Tween^®^20.

### 2.3. Characterization of the Nanoprobes

Ultraviolet–visible (UV-Vis) absorption measurements were taken using a Tecan Infinite M Nano plate reader. Zeta-potential (ζ-potential) and dynamic light scattering (DLS) measurements of nano-particles and probes were obtained using a Malvern Zetasizer at 25 °C. The concentration of DNA hairpins attached to the AuNPs was determined by measuring the absorbance of the reacted supernatant using a NanoDrop ONE spectrophotometer (Thermo Fisher Scientific, Waltham, MA, USA)

### 2.4. Fabrication of Paper-Based Lateral Flow System and Sample Setup

The lateral flow strips used in this work consisted of a test membrane and a wicking pad, both affixed to an adhesive card. The wicking pads were composed of CF7 paper (100% cotton linter, 1873 μm, Whatman, Marlborough, MA, USA, CYTIVA), while the test membrane was composed of nitrocellulose (FF120 HP, Whatman, CYTIVA). To prepare the test line, we applied 500 μL of a pre-mixed aqueous solution containing 1.25 mM streptavidin and 125 μM biotinylated capture DNA hairpins. For the control line, we used 500 μL of a solution with 0.625 mM streptavidin and 125 μM biotinylated control DNA hairpins. Both lines were deposited using a syringe pump connected to a lateral flow reagent dispenser (Claremont Bio, Upland, CA, USA) at a flow rate of 0.2 mL/min, producing lines approximately 1 mm wide. After deposition, the paper was cut into 5 mm × 40 mm test strips.

To evaluate the specificity of the test, we prepared samples containing 50 μL of hsa-miR-20a-5p, which has a two-base mismatch with the target sequence. Positive control samples contained 50 μL of hsa-miR-17-5p at varying concentrations. For lateral flow assay (LFA) testing, we deposited the samples into the wells of a 96-well plate. We then added 100 μL of DNA-SiO_2_-AuNS particles (optical density 0.4) diluted in 2× SSPE buffer containing 3% Tween^®^ 20 to each well. After a 30 min incubation, lateral flow strips were placed into the wells to allow the development of test and control lines. All tests were completed within 30 min, and colorimetric and SERS measurements were conducted immediately afterward.

### 2.5. Quantification of SERS and Colorimetric Signals on the LFA System

The portable Raman system used for SERS analysis consisted of a WP-785-R-SR-LMMFC-25 spectrometer (Wasatch Photonics, Logan, UT, USA) with an integrated diode laser (450 mW, 785 nm) connected to an RP-785 Raman probe (Wasatch Photonics). Three spectra were collected in each region (i.e., control line, test line, and blank region between the test and control lines) using a 1 s integration time and a power of 21 mW. The z-position (aperture-to-sample distance) was fixed throughout all experiments at 11 mm. Prior to analysis of the Raman spectra, baseline correction was performed using the asymmetric least squares smoothing method [24] in × R2023b.

For colorimetric measurements, lateral flow strips were imaged using an iPhone 11 equipped with a dual 12 megapixel wide and ultra-wide camera. Prior to collecting images, the test strips were placed on a sheet of white paper to enhance contrast and aid visualization. The paper strips were arranged side by side to minimize light-source scattering, facilitating equitable comparison of results. Images captured in JPEG format were processed via ImageJ V1.54h (NIH, Bethesda, MD, USA) using the RGB measure plugin after pre-processing the images to normalize their contrast. Three different regions of interest were used for quantification, namely, test line, control line, and blank regions (the area between the control and test lines).

### 2.6. Preparation and Extraction of Cell-Free miRNAs from Spiked Human Serum Samples

Pregnancy-specific human serum samples were purchased from Discovery Life Science, and synthetic hsa-miR-17-5p was obtained from IDT. To create the spiked samples, we added 2 μL of hsa-miR-17-5p at concentrations ranging from 0.15 to 1500 pg/μL to 198 μL of each serum sample, achieving a 100-fold dilution. Total cell-free RNA, including microRNAs, was isolated using the QIAGEN miRNeasy Advanced kit. In brief, the spiked serum samples were mixed with 60 μL of RPL buffer, vortexed for 20 s, and incubated at room temperature for 3 min. Next, 20 μL of RPP buffer was added to each sample; this was followed by vortexing for 20 s and incubation at RTP for 5 min. The samples were then centrifuged at 10,000 rpm for 3 min. The supernatant from each sample was transferred to an RNeasy UCP MinElute column and centrifuged at 8000 rpm for 1 min. After discarding the flow-through, 700 μL of RWT buffer was added to each column. This washing process was repeated twice, first with 500 μL of RPE buffer and then with 500 μL of 80% ethanol, each time followed by centrifugation and discarding of the flow-through. Finally, the column was dried, and the RNA was eluted into 20 μL of nuclease-free water.

### 2.7. Reverse Transcription Quantitative Polymerase Chain Reaction (RT-qPCR) of hsa-miR-17-5p Levels in Serum Samples

Reverse transcription quantitative PCR (RT-qPCR) was performed as a quality control step using TaqMan^®^ miRNA assays to quantify the recovery of hsa-miR-17-5p from each serum sample. For each RT reaction, a mixture was prepared, containing 5 µL of RNA extract, 3 µL of stem-loop primer, 0.15 µL of 100 mM dNTPs, 1 µL of MultiScribe™ reverse transcriptase (50 U/µL), 1.5 µL of 10× reverse transcription buffer, 0.19 µL of RNase inhibitor (20 U/µL), and 4.16 µL of nuclease-free water, bringing the total volume to 15 µL. This mixture was incubated sequentially at 16 °C for 30 min, 42 °C for 30 min, and 85 °C for 5 min and then stored at 4 °C until the qPCR step. For qPCR, each 10 µL reaction mixture contained 0.5 µL of 20× TaqMan™ Small RNA Assay probe, 5 µL of TaqMan™ Fast Advanced Master Mix, 3.84 µL of nuclease-free water, and 0.67 µL of cDNA synthesized from the RT step. The qPCR was carried out using a BioRad CFX384 real-time PCR detection system. After an initial denaturation at 95 °C for 20 s, 40 cycles of amplification were performed, each consisting of denaturation at 95 °C for 3 s followed by annealing and extension at 60 °C for 30 s. Quantification was achieved by determining the quantification cycle (Cq), using 2000 relative fluorescence units (RFU) as the baseline fluorescence threshold.

### 2.8. Statistical Analysis of RT-qPCR, Colorimetric, and SERS-Based Measurements Using the Statistical Package for Social Science (SPSS)

Sample sizes for each experiment are indicated in their respective figures. We plotted variables—including the quantification cycle (Cq) values from RT-qPCR, colorimetric measurements (RGB intensity), and SERS intensities at 1080 cm^−1^ and 1580 cm^−1^—across different concentrations of hsa-miR-17-5p in water aliquots and spiked serum samples. To determine whether the data were normally distributed and had equal variances among groups, we applied the Shapiro–Wilk test and Levene’s test, respectively. One-way ANOVA tests were performed using SPSS Statistics 29 (IBM, Armonk, NY, USA). For pairwise comparisons in the ANOVA, we used Tukey’s Honestly Significant Difference post hoc test, with a significance level of α = 0.001.

## 3. Results and Discussion

**Figure 1:**
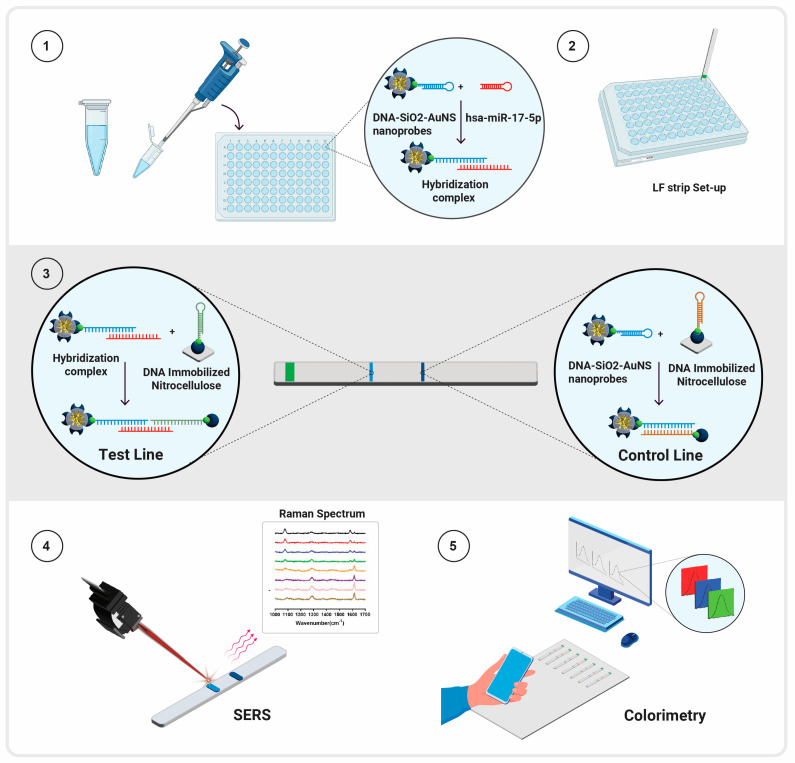
Schematic diagram illustrating the workflow of the SERS-based lateral flow assay (LFA) used for the quantification of hsa-miR-17-5p. In Step 1, the sample spiked with hsa-miR-17-5p and the nanoprobe was incubated in a test tube for up to 15 min. In Step 2, the mixture of the sample and nanoprobe was transferred to a 96-well plate, and the LFA system was introduced into the well containing the mixture. In Step 3, the development of the test and control lines occurred within 15 min via natural capillary action. In Step 4, surface-enhanced Raman scattering (SERS) spectra were captured and analyzed using a Raman spectrometer. In Step 5, images of the LFA assay were taken using a smartphone, and ImageJ V1.54h software was employed to perform colorimetric analysis.

The assay workflow is illustrated in Figure 1. Step 1 shows the mixing of DNA-SiO_2_-AuNS probes with the miRNA target in a well of a plate, where it was incubated for 30 min to enable the formation of the hybridization complex. After incubation, the LF strip was inserted into the well (Step 2). The sandwich assay complexes formed at the test and control lines on the LF strip are shown in Step 3. After the test was complete, SERS spectra were collected from the strip using a portable fiber-optic Raman spectrometer (Step 4), and the images used for RGB analysis were collected using a smart phone (Step 5).

### 3.1. Validating the Capture and Detector of the miRNA Using Optimized DNA Hairpin Sequences

DNA hairpin probes are highly versatile nucleic acid probes that have been extensively utilized in the development of biosensors, both in solid-state-based platforms [25] and aqueous or homogeneous systems [26]. Their unique structures and ability to undergo conformational changes make them particularly suited for use in lateral flow assays, where their high specificity and sensitivity can enhance detection performance [27]. To ensure that the targeting of the hsa-miR-17 using the DNA hairpins was specific, we first simulated the sandwich assay formations using a variety of hairpin candidates. Hybridization reactions were simulated using Nucleic Acid Package (NUPACK 4.0) [28] software to identify the best hairpin sequences for targeting hsa-miR-17-5p by screening 40 different candidates under varying temperatures (4–95 °C). The bank of hairpin candidates was created by varying the lengths and sequences of the stem and loop regions between 3 to 6 base pairs. The resultant Gibb’s free energy and estimated concentration of the desired sandwich products was used to rank the candidates. Our goal was to select a capture and detector hairpin probe pair that could be used to selectively target the miRNA while limiting cross-hybridization.

Among the 40 candidates (of which the 38 unchosen sequences are listed in Supplementary Table S1), the best candidates for the detector and capture hairpins are listed in Table 1. The Gibbs free energy values for the detector and capture hairpins were −3.70 kcal/mol and −2.31 kcal/mol at 37 °C, suggesting that neither hairpin was identified as a major contributor to the formation of unwanted hybridizations. The secondary structures of sandwich triplex, target, detector, and capture hairpins are shown in Supplementary Figure S1A–D.

**Figure 2:**
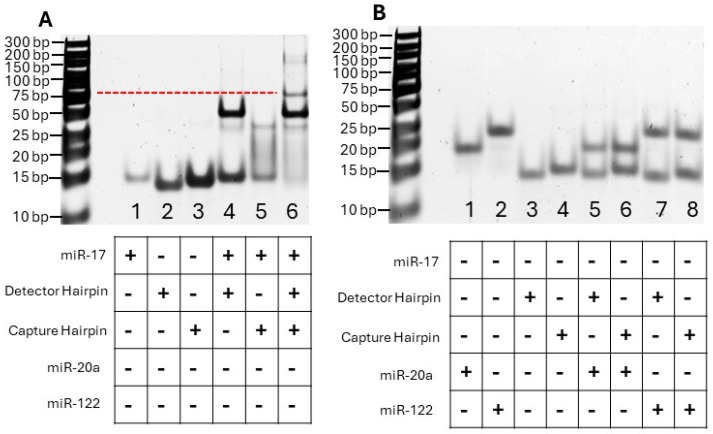
Image of 15% native PAGE gel for validation of DNA-RNA hybridization between DNA hairpins and hsa-miR-17-5p. ‘+’ means the presence of a given species in a lane, while ‘-’ indicates the absence of that species in the lane. A total of 150 pg/µL of the sequences was added to each lane. (**A**) A gel image indicating the desired binding between dual DNA-hairpin probes and hsa-miR-17-5p. (**B**) A gel image indicating no non-specific binding between dual DNA-hairpin probes and other non-specific targets (has-miR-20a-5p, 2 nt mismatched; has-miR-122-5p, fully mismatched). Red dashed line indicates the referred position of the desired sandwich product.

To experimentally demonstrate the hybridization affinity between the selected detector and capture hairpins and the target, we performed a standard native PAGE analysis. The images are shown in Figure 2A. As both the detector and capture hairpins are 22 nt in length and the target sequence is 23 nt long, the bands appear in the gel at similar positions as observed in lanes 1–3, respectively. As expected, higher-molecular-weight bands were observed in lane 4, when the detector hairpin and target were hybridized, and in lane 5, when the capture hairpin and target were combined, suggesting that the hairpins had successfully bound to the target. When the detector and capture hairpins were combined with the target in lane 6, a third band at a higher molecular weight could be observed, which suggests successful duplex formation. However, while the desired hybridization complex was being formed, the presence of two additional bands at the top of lane 6 suggested non-specific leakage products were being formed, which is common in molecular amplifications. This leakage was tentatively attributed to high-order complex formation between the DNA conjugated to the surface of the SiO_2_-AuNS particles and hsa-miR-17-5p.

To test the specificity of the hairpins, we also examined hybridizations using standard native PAGE, which included the use of two control sequences, miR-20a-5p and miR-122-5p, which feature two or more base pair mismatches, respectively (the sequences are given in Table 1). The gel is shown in Figure 2B. Lanes 1–4 show the bands of hsa-miR-20a-5p, hsa-miR-122-5p, and the detector and capture hairpins. The bands that could be attributed to greater-molecular-weight products were absent in Lane 5–8, implying that non-specific hybridization between the detection and capture hairpins was not occurring in the presence of the control sequences.

### 3.2. Characterization of Nanoprobes

**Figure 3:**
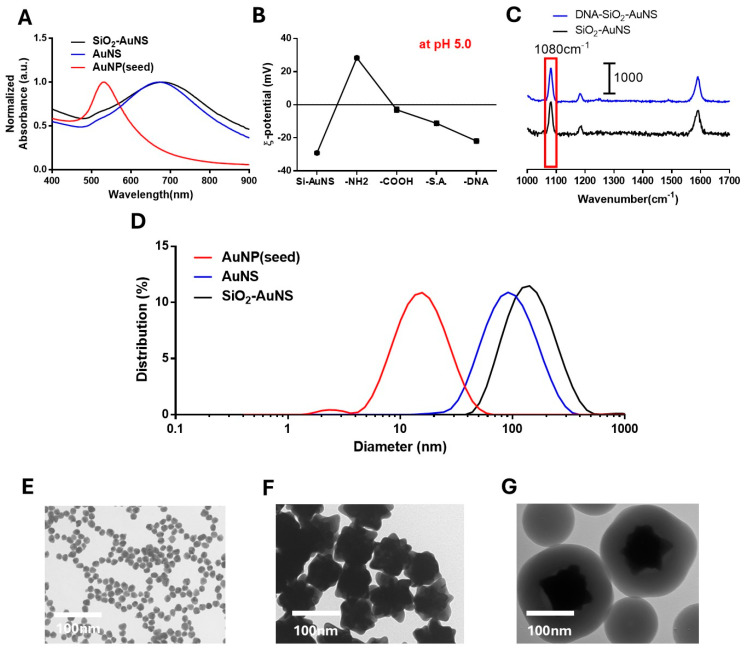
Characterization of DNA/Silica-coated nanostar (DNA-SiO_2_-AuNS). (**A**) UV-Vis spectra of AuNP(seed), AuNS, and SiO_2_-AuNS; (**B**)  ζ-potential of SiO_2_-AuNS throughout the functionalization of SiO_2_-AuNS; (**C**) normalized SERS signal comparison before and after DNA functionalization; (**D**) DLS analysis of the diameters of AuNP, AuNS, and SiO_2_-AuNS; (**E**) TEM image of AuNP (seed); (**F**) TEM image of AuNS; (**G**) TEM image of SiO_2_-AuNS.

Figure 3A shows the UV-Vis spectra representative of the AuNPs, AuNSs, and SiO_2_-AuNS nanoprobes. The lambda max (λ_max_) values for the particles were 523, 620 nm, and 670 nm, respectively. A shift from 523 nm to 620 nm was expected since the AuNPs are red, whilst the AuNS nanoprobes are blue. Furthermore, the shift from 620 nm to 670 nm observed upon coating the AuNS particles with silica was expected due to the increased refractive index provided by the silica shell. The results of the DLS analysis given in Figure 3D show that the median diameters of AuNP, AuNS, and SiO_2_-AuNS were approximately 11 nm, 100 nm, and 170 nm, respectively. Particle size was estimated from the TEM images collected for each type of particle; the data for AuNPs (Figure 3E), AuNS (Figure 3F), and SiO_2_-AuNS (Figure 3G) are consistent with the DLS data.

The successful conjugation of the DNA hairpins to the surface of the SiO_2_-AuNS nanoprobes is evidenced by the change in the ζ-potential of the products after each functionalization step (Figure 3B). The nanoprobes initially had a surface potential of ~35 mV. However, after surface modification via APTES, the charge expectedly increased to +30 mV due to the presence of primary amine groups. Conversion of the amine groups to carboxylic acid groups in the subsequent step induced a negative surface charge, resulting in a drop in ζ-potential to −5 mV. Finally, as streptavidin and DNA are both negatively charged at pH 5, a further decrease in ζ-potential occurred after bioconjugation.

SERS measurements of the nanoprobes were taken before and after functionalization with DNA (Figure 3C). We observed that the Raman peaks specific to 4-MBA, at 1080 cm^−1^ and 1580 cm^−1^, were present in both DNA-SiO_2_-AuNS and SiO_2_-AuNS, showing that the SERS signal expectedly remained stable throughout the process. This result aligns with those from other studies characterizing the interactions between 4-MBA-modified gold surfaces [29]. Additionally, the subsequent addition of the silica coating and the functionalized DNA did not hinder SERS analysis of the 1080 cm^−1^ and 1580 cm^−1^ peaks corresponding to 4-MBA.

### 3.3. Validation of hsa-miR-17-5p Levels in Spiked Serum Samples Using RT-qPCR

While there is a certain abundance of cell-free miRNAs in maternal serum samples, at around 219 to 844 fM (~1.5 pg/µL to 6.4 pg/µL) [30], targeting them directly without extraction is difficult since they either conjugate with argonaut proteins to form an miRNA–argonaut complex [31,32] or become encapsulated in extracellular vesicles [33] and exosomes [34]. Therefore, a standardized extraction method must be employed to make sure the miRNA becomes accessible for their accurate quantification.

To simulate the need for an extraction process and determine the recovery, we spiked a known amount of hsa-miR-17-5p or hsa-miR-20a-5p into human serum and then proceeded with total cell-free RNA extraction using the miRNeasy Advanced extraction kit. This kit was specifically selected since it corresponds to a phenol-free method. This is important since phenolic compounds display prominent peaks at ~1208 cm^−1^ and ~1602 cm^−1^ that would interfere with our ability to quantify the hsa-miR-17-5p sequence based on our SERS spectral analysis.

**Figure 4:**
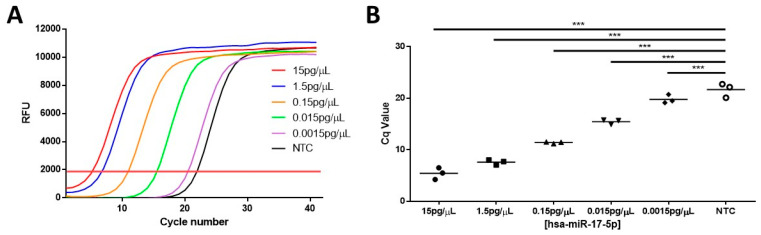
hsa-miR-17-5p detection in the spiked serum sample after extraction with QIAGEN miRNeasy Advanced Kit utilizing the Taqman^®^ stem-loop miRNA assay. (**A**) Representative real-time fluorescent curve with different concentrations of hsa-miR-17-5p spiked in the serum sample. (**B**) Average and individual Cq values of each group (n = 3). NTC is normal female serum with stratified age, gestational week, and ethnicity. (***: α ≤ 0.001).

The RT-qPCR data displayed in Figure 4A show that there is an inverse correlation between spiked hsa-miR-17-5p concentrations and the cycles of quantification (Cq) values across expected physiological concentrations ranging from 15 pg/µL to 0.0015 pg/µL. It was observed that the Cq at the lowest concentration of 0.0015 pg/µL was similar to the Cq for the no-template control (NTC) group, thus defining our lowest observed concentration (Figure 4B). This possibly implies that the sera contained a base level of hsa-miR-17-5p prior to spiking.

### 3.4. Measured SERS Intensity of hsa-miR-17-5p Levels in Water Aliquot of hsa-miR-17-5p and Spiked Serum Samples

**Figure 5:**
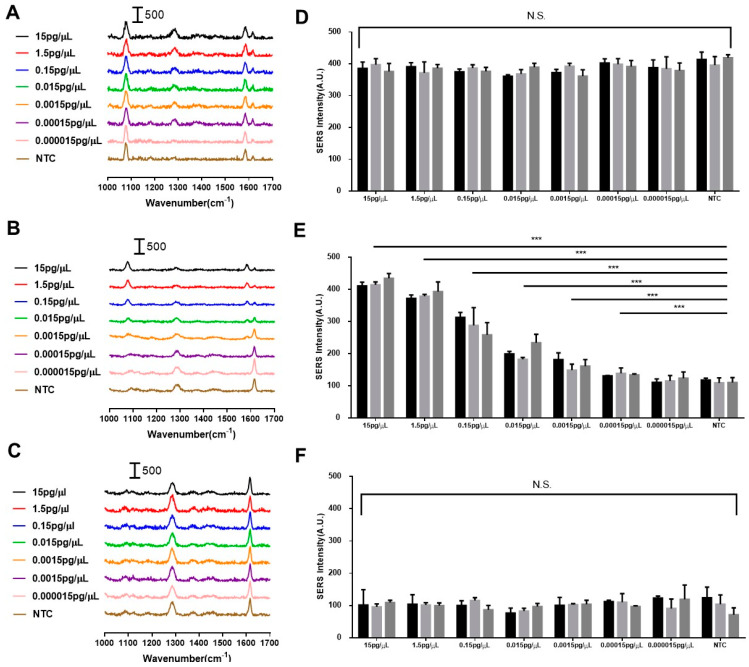
SERS-based analysis on paper-based LFA targeting hsa-miR-17-5p in aliquots of different concentrations of hsa-miR-17-5p in nuclease-free water. (**A**) Normalized SERS spectra captured from the control line; (**B**) normalized SERS spectra captured from the test line; (**C**) SERS spectra captured from the blank regions; (**D**) SERS intensity at 1080 cm^−1^ on control line; (**E**) SERS intensity at 1080 cm^−1^ on test line; (**F**) SERS intensity at 1080 cm^−1^ in blank regions (*** α ≤ 0.001). N.S. refers to not statistically significant.

**Figure 6:**
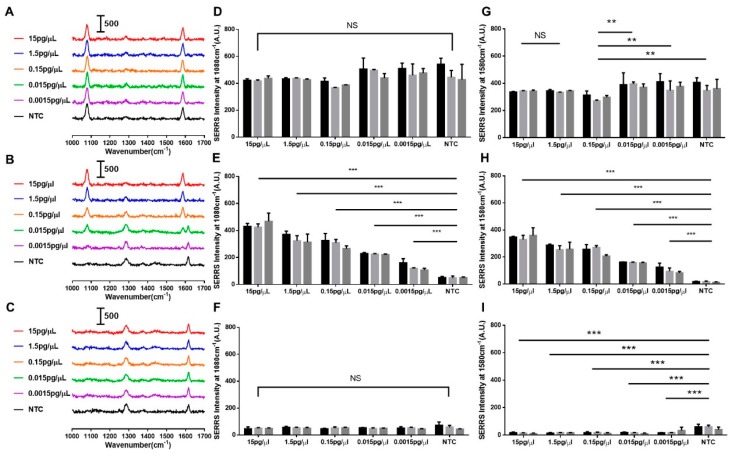
SERS-based analysis on paper-based LFA targeting hsa-miR-17-5p in spiked samples of different concentrations of hsa-miR-17-5p. (**A**) Normalized SERS spectra captured from the control line; (**B**) normalized SERS spectra captured from the test line; (**C**) SERS spectra captured from the blank regions; (**D**) SERS intensity at 1080 cm^−1^ on control line; (**E**) SERS intensity at 1080 cm^−1^ on test line; (**F**) SERS intensity at 1080 cm^−1^ on blank regions; (**G**) SERS intensity at 1580 cm^−1^ on control line; (**H**) SERS intensity at 1580 cm^−1^ on test line; (**I**) SERS intensity at 1580 cm^−1^ on blank regions (*** α ≤ 0.001,** α ≤ 0.05). NS refers to not statistically significant.

The capture of the SERS intensity was guided by the resultant control line and test line. Colorimetric results from both the aliquot and spiked serum samples can be found in Supplementary Figures S2 and S3, with a limit of detection (LOD) of 0.7802 pg/µL in spiked serum samples, as estimated using the methodology outlined by Armbruster et al. [31]. The SERS intensities of the test and control line regions were measured. We also captured SERS spectra from a blank region situated between the test and control lines to elucidate the baseline SERS signal observed where there were no capture reagents. The DNA-SiO_2_-AuNS exhibited two prominent peaks at 1080 cm^−1^ and 1580 cm^−1^, observable at both the control and test lines, particularly at elevated concentrations of hsa-miR-17-5p. An intriguing observation was the appearance of the 1620 cm^−1^ peak that was attributed to nitrocellulose; it was inversely correlated with the 1580 cm^−1^ peak, as the concentration of hsa-miR-17-5p decreased, as shown in Figure 5A–C and Figure 6A–C. We hypothesized that this phenomenon was due to the area coverage of the nanoprobes on the nitrocellulose. Notably, the SERS intensity of the 1080 cm^−1^ peak measured at the control line remained stable, with no statistical significance (Figure 5A,D and Figure 6A,D), whereas in Figure 6G, the SERS intensity of the 1580 cm^−1^ peak measured at the control line showed some minor differences between the NTC group and other groups (α ≤ 0.05). Hence, the 1080 cm^−1^ peak was used as the optimal vibrational mode to aid in distinguishing hsa-miR-17-5p concentrations.

In this preliminary study, we first validated if the LFA device could be sensitive to only hsa-miR-17-5p without interference from other miRNA species that may naturally exist in spiked serum samples. Therefore, SERS intensity was first measured using LFA devices, a process that began with a water aliquot of hsa-miR-17-5p. In Figure 5A,C, the SERS intensities in the control line and blank regions remain the same. In addition, no significant differences could be found between the control line and blank regions of different concentrations of hsa-miR-17-5p at the 1080 cm^−1^ peak, as illustrated in Figure 5D,F. However, we observed a clear gradient decrease in SERS intensity at the 1080 cm^−1^ peak, with a reduction in the concentration of hsa-miR-17-5p in the aliquot samples (Figure 5B). In particular, as illustrated in Figure 5E, there were statistically significant differences in the mean SERS signal intensities of all the other groups, except the 0.000015 pg/µL group, when compared with the NTC (α ≤ 0.001). This validation ensures that the LFA device can detect hsa-miR-17-5p aliquoted in water.

**Figure 7:**
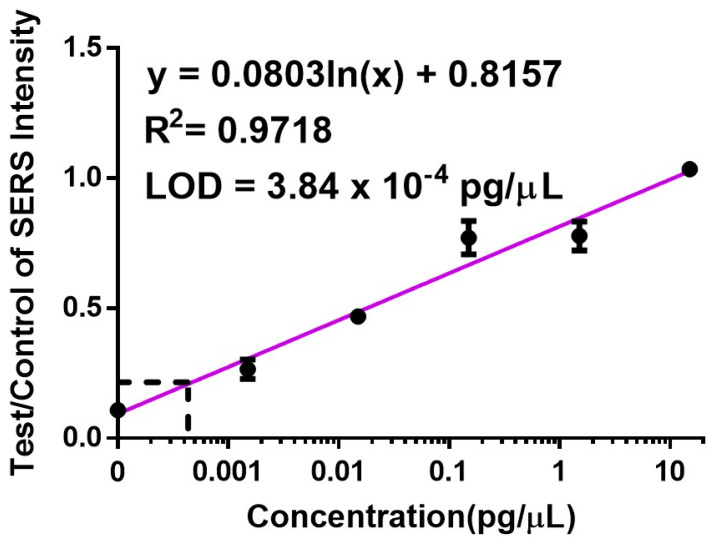
SERS LOD calculation. The calibration curve shows the ratio of the SERS signal at 1080 cm^−1^ obtained from the test and control lines for samples with varying concentrations of hsa-miR-17-5p.

Then, we further proceeded with using the spiked serum sample on the LFA system. Spectra collected from the test line (Figure 6E,H) show that the intensity of both the 1080 cm^−1^ and 1580 cm^−1^ peaks correlates with hsa-miR-17-5p concentrations, providing statistically significant differences in the mean SERS signal intensity when compared with the NTC (α ≤ 0.001). In Figure 6H,I, the blank region displays a minimal SERS background signal, implying that the nanoprobes flowed through the nitrocellulose membrane towards the test and control lines unhindered. Preliminary experiments where hsa-miR-17-5p was spiked in water samples (Supplementary Figure S3D–F) displayed similar trends to those observed in Figure 6D–F. Finally, for quantification purposes, the ratio of SERS intensities for the 1080 cm^−1^ peaks were analyzed. As illustrated in Figure 7, we calculated the LOD for the SERS readout in spiked serum samples to be 3.84 × 10^−4^ pg/µL using the the same method [35]. Upon comparing the LODs in our assay, it becomes evident that the SERS analysis (LOD = 3.84 × 10^−4^ pg/µL) was approximately 2000 times more sensitive than the colorimetric method (LOD = 0.7802 pg/µL). This finding confirms that our approach consisting of using an LFA assay with our nanoprobes for the detection of hsa-miR-17-5p would be efficacious for the sensitive and accessible quantification of miRNA at sub-picomolar levels.

## 4. Conclusions

We have successfully developed an LFA assay by combining hairpin-based detector and capture sequences for the specific capture of hsa-miR-17-5p, a potential biomarker for preeclampsia screening, with a novel design of DNA hairpins and nanoprobes. By employing a portable Raman spectrometer to capture SERS spectra and utilizing selected DNA hairpins specifically designed to capture hsa-miR-17-5p, we have demonstrated the feasibility of the LFA assay for detecting miRNA in human serum post-extraction via SERS, which was validated using RT-qPCR. The SERS analysis revealed a significantly low LOD of 3.84 × 10^−4^ pg/µL, reaching a level comparable to that of RT-qPCR with respect to capturing has-miR-17-5p in spiked serum samples. This study demonstrates that integrating SERS analysis into the LFA assay can provide significantly better sensitivity compared to that of colorimetry for detecting circulating miRNA while simultaneously using DNA hairpins as capture ligands to achieve high specificity.

## Figures and Tables

**Table 1:** DNA or RNA sequences of detector hairpin on SiO_2_-AuNS and capture sequences on test line and control line, hsa-miR-17-5p, hsa-miR-20a-5p, and hsa-miR-122-5p. Differences in nucleotides between hsa-miR-17-5p and hsa-miR-20a-5p are highlighted in red.

Name	Sequence (5′ to 3′)
Detector Hairpin	/5BioTEG/AAAAAAAAAAAGGTCTACCTGCA
Capture Hairpin	CTGTAGCACTTTGCTAAAAAAA/3BioTEG/
Control Line Capture Hairpin	TGCAGGTAGCCTTTTTTTTTTT/3BioTEG/
hsa-miR-17-5p (Target)	CAAAGUGCUACAGUGCAGGUAGU
hsa-miR-20a-5p	UAAAGUGCUUCAGUGCAGGUAGU
hsa-miR-122-5p	UGGAGUGUGACAAUGGUGUUUG

## Data Availability

The data presented in this study are available on reasonable request from the corresponding author.

## Supplementary Materials

The following supporting information can be downloaded at https://www.mdpi.com/article/10.3390/bios14110535/s1.
