# Supplementary material for: Lateral Flow Assay for Preeclampsia Screening Using DNA Hairpins and Surface-Enhanced Raman-Active Nanoprobes Targeting hsa-miR-17-5p
Source: Biosensors (Basel). 2024 Nov 5;14(11):535. doi: 10.3390/bios14110535 (PMC11592307; doi:10.3390/bios14110535)
Supplement: Supplementary file 1 [file biosensors-14-00535-s001.zip › biosensors-3214564-supplementary.pdf]

Supplementary Information

Lateral Flow Assay for Preeclampsia Screening Using DNA Hairpins and Surface-Enhanced Raman-Active Nanoprobes Targeting hsa-miR-17-5p

Ka Wai Ng [1,†], Siddhant Jaitpal [1,†], Ngoc Nhu Vu [1], Angela Michelle T. San Juan [1], Sayantan Tripathy [1], Rohit Sai Kodam [1], Abhishek Bastiray [2], Jae-Hyun Cho [2], Mahua Choudhury [3], Gerard L. Coté [1,4,5] and Samuel Mabbott [1,4,*]

[1] Department of Biomedical Engineering, Texas A&M University, 5045 Emerging Technologies Building, College Station, TX 77843, USA
[2] Department of Biochemistry and Biophysics, Texas A&M University, College Station, TX 77843, USA
[3] Irma Lerma Rangel College of Pharmacy, Texas A&M University, 159 Reynolds Medical Building, College Station, TX 77843, USA
[4] Center for Remote Health Technologies & Systems, Texas A&M Engineering Experimentation Station, 1041 Emerging Technologies Building, College Station, TX 77843, USA
[5] Department of Electrical Engineering, Texas A&M University, College Station, TX, 77843, USA
* Correspondence: smabbott@tamu.edu
† These authors contributed equally to this work.

Supplementary Table S1. Other possible candidates of DNA sequences for probe functionalized on SiO_2-AuNS, capture sequences on test line.

Name	Sequence (5' to 3')
Detector Hairpin_1	/5BioTEG/AAAAAAAAAACAGGCTACCTGCA
Detector Hairpin_2	/5BioTEG/AAAAAAAAAAGCAGGCTACCTGCA
Detector Hairpin_3	/5BioTEG/AAAAAAAAAATGCAGGCTACCTGCA
Detector Hairpin_4	/5BioTEG/AAAAAAAAAAAGGACTACCTGCA
Detector Hairpin_5	/5BioTEG/AAAAAAAAAACAGGACTACCTGCA
Detector Hairpin_6	/5BioTEG/AAAAAAAAAAGCAGGACTACCTGCA
Detector Hairpin_7	/5BioTEG/AAAAAAAAAATGCAGGACTACCTGCA
Detector Hairpin_8	/5BioTEG/AAAAAAAAAAAGGAACTACCTGCA
Detector Hairpin_9	/5BioTEG/AAAAAAAAAACAGGAACTACCTGCA
Detector Hairpin_10	/5BioTEG/AAAAAAAAAAGCAGGAACTACCTGCA
Detector Hairpin_11	/5BioTEG/AAAAAAAAAATGCAGGAACTACCTGCA
Detector Hairpin_12	/5BioTEG/AAAAAAAAAAAGGAAACTACCTGCA
Detector Hairpin_13	/5BioTEG/AAAAAAAAAACAGGAAACTACCTGCA
Detector Hairpin_14	/5BioTEG/AAAAAAAAAAGCAGGAAACTACCTGCA
Detector Hairpin_15	/5BioTEG/AAAAAAAAAATGCAGGAAACTACCTGCA
Detector Hairpin_16	/5BioTEG/AAAAAAAAAAAGGAAAACTACCTGCA
Detector Hairpin_17	/5BioTEG/AAAAAAAAAACAGGAAAACTACCTGCA
Detector Hairpin_18	/5BioTEG/AAAAAAAAAAGCAGGAAAACTACCTGCA
Detector Hairpin_19	/5BioTEG/AAAAAAAAAATGCAGGAAAACTACCTGCA
Capture Hairpin_1	CTGTAGCACTTTGCTAAAGAAAA/3BioTEG/
Capture Hairpin_2	CTGTAGCACTTTGCTAAAGTAAAA/3BioTEG/
Capture Hairpin_3	CTGTAGCACTTTGCTAAAGTGAAAA/3BioTEG/
Capture Hairpin_4	CTGTAGCACTTTGCTAAAAAAAA/3BioTEG/
Capture Hairpin_5	CTGTAGCACTTTGCTAAAAGAAAA/3BioTEG/
Capture Hairpin_6	CTGTAGCACTTTGCTAAAAGTAAAA/3BioTEG/
Capture Hairpin_7	CTGTAGCACTTTGCTAAAAGTGAAAA/3BioTEG/
Capture Hairpin_8	CTGTAGCACTTTGCTAAAAAAAAA/3BioTEG/
Capture Hairpin_9	CTGTAGCACTTTGCTAAAAAGAAAA/3BioTEG/
Capture Hairpin_10	CTGTAGCACTTTGCTAAAAAGTAAAA/3BioTEG/
Capture Hairpin_11	CTGTAGCACTTTGCTAAAAAGTGAAAA/3BioTEG/
Capture Hairpin_12	CTGTAGCACTTTGCTAAAAAAAAAA/3BioTEG/
Capture Hairpin_13	CTGTAGCACTTTGCTAAAAAAGAAAA/3BioTEG/
Capture Hairpin_14	CTGTAGCACTTTGCTAAAAAAGTAAAA/3BioTEG/
Capture Hairpin_15	CTGTAGCACTTTGCTAAAAAAGTGAAAA/3BioTEG/
Capture Hairpin_16	CTGTAGCACTTTGCTAAAAAAAAAAA/3BioTEG/
Capture Hairpin_17	CTGTAGCACTTTGCTAAAAAAAGAAAA/3BioTEG/
Capture Hairpin_18	CTGTAGCACTTTGCTAAAAAAAGTAAAA/3BioTEG/
Capture Hairpin_19	CTGTAGCACTTTGCTAAAAAAAGTGAAAA/3BioTEG/

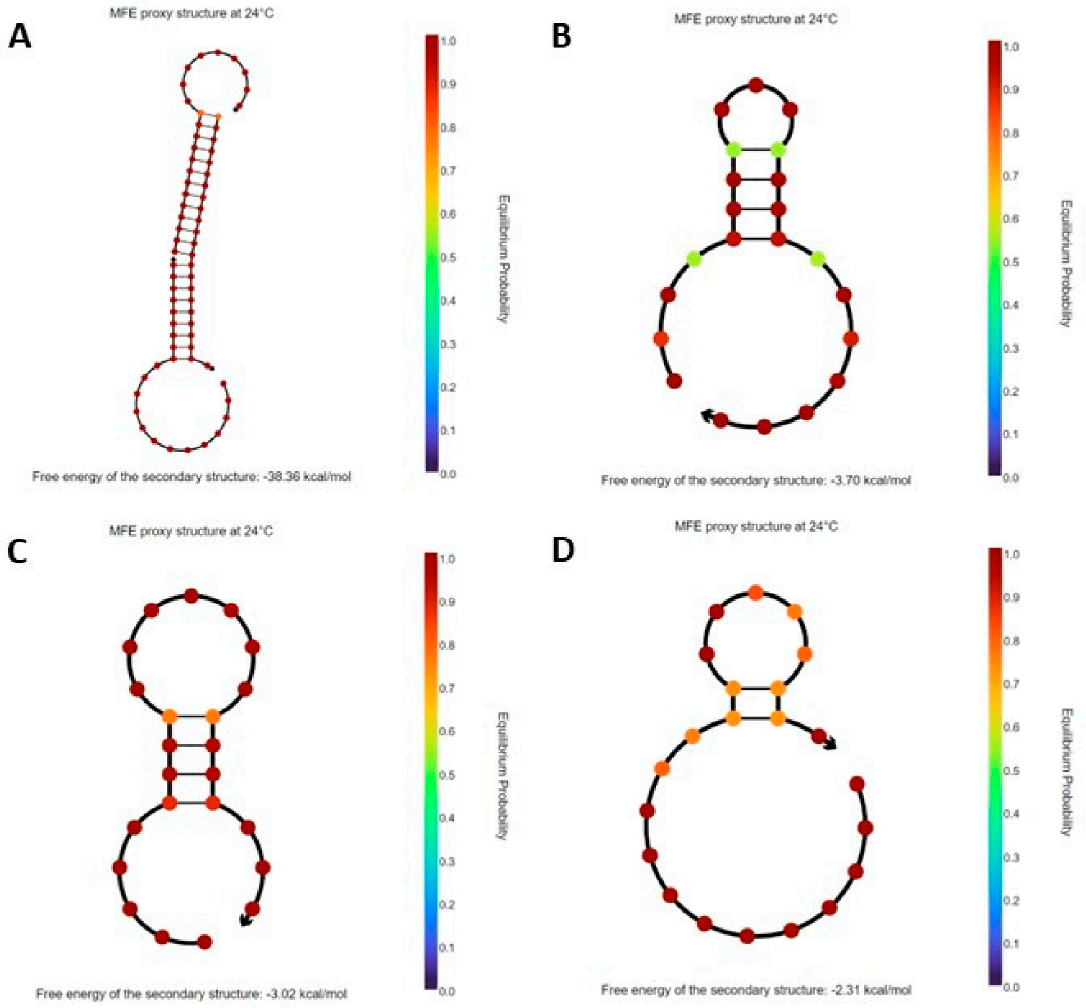

Supplementary Figure S1. Secondary structures of minimum free energy ensemble at 37ºC by NUPACK modeling of the selected candidates. A. Predicted secondary structure of hybridized sandwich product; B. Predicted secondary structure of recognition hairpin; C. Predicted secondary structure of hsa-miR-17-5p; D. Predicted secondary structure of capture hairpin.

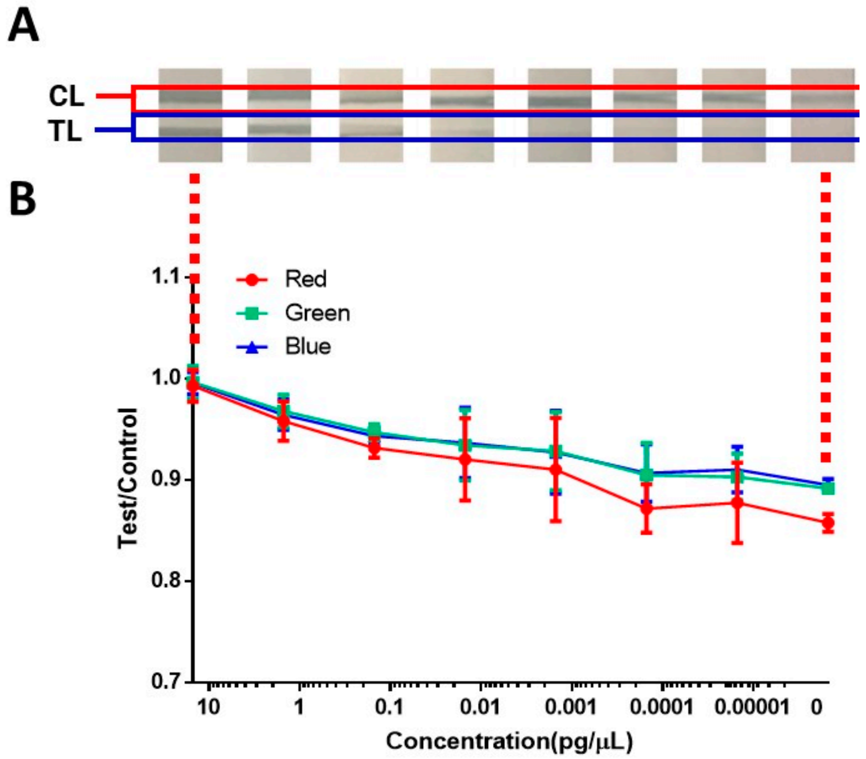

Supplementary Figure S2. Colorimetric analysis on paper-based LFA targeting hsa-miR-17-5p in water aliquot; A: Result photos of LFA after spiking in different samples containing different concentrations of target(15 pg/µL, 1.5 pg/µL, 0.15 pg/µL,); B: The red, green and blue intensities of test area on strips for detecting different concentrations of hsa-miR-17-5p using DNA-SiO_2-AuNS, respectively. Each data point represents the mean (±standard deviation) of triplicate experiments, and the intersections of two-dotted lines label the LODs on each.

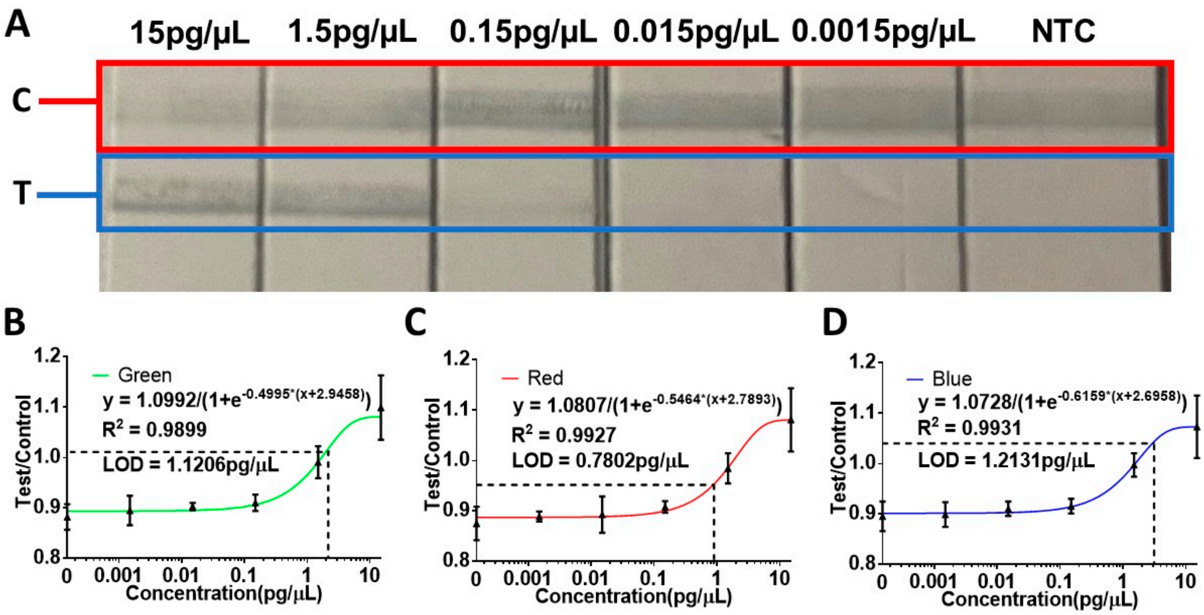

Supplementary Figure S3. Colorimetric analysis on the LFA device targeting hsa-miR-17-5p in spiked samples; A: Images of LFA after spiking in different samples containing different concentrations of target(15 pg/μL, 1.5 pg/μL, 0.15 pg/μL, 0.015 pg/μL, 0.0015 pg/μL, NTC); B. Ratio of green intensities of test line area versus control line area on strips for detecting different concentrations of hsa-miR-17-5p using DNA-SiO_2-AuNS; C. Ratio of red intensities of test line area versus control line area on strips for detecting different concentrations of hsa-miR-17-5p using DNA-SiO_2-AuNS; D. Ratio of blue intensities of test line area versus control line area on strips for detecting different concentrations of hsa-miR-17-5p using DNA-SiO_2-AuNS Each data point represents the mean (±standard deviation)of triplicate experiments, and the intersections of two-dotted lines label the LODs on each.

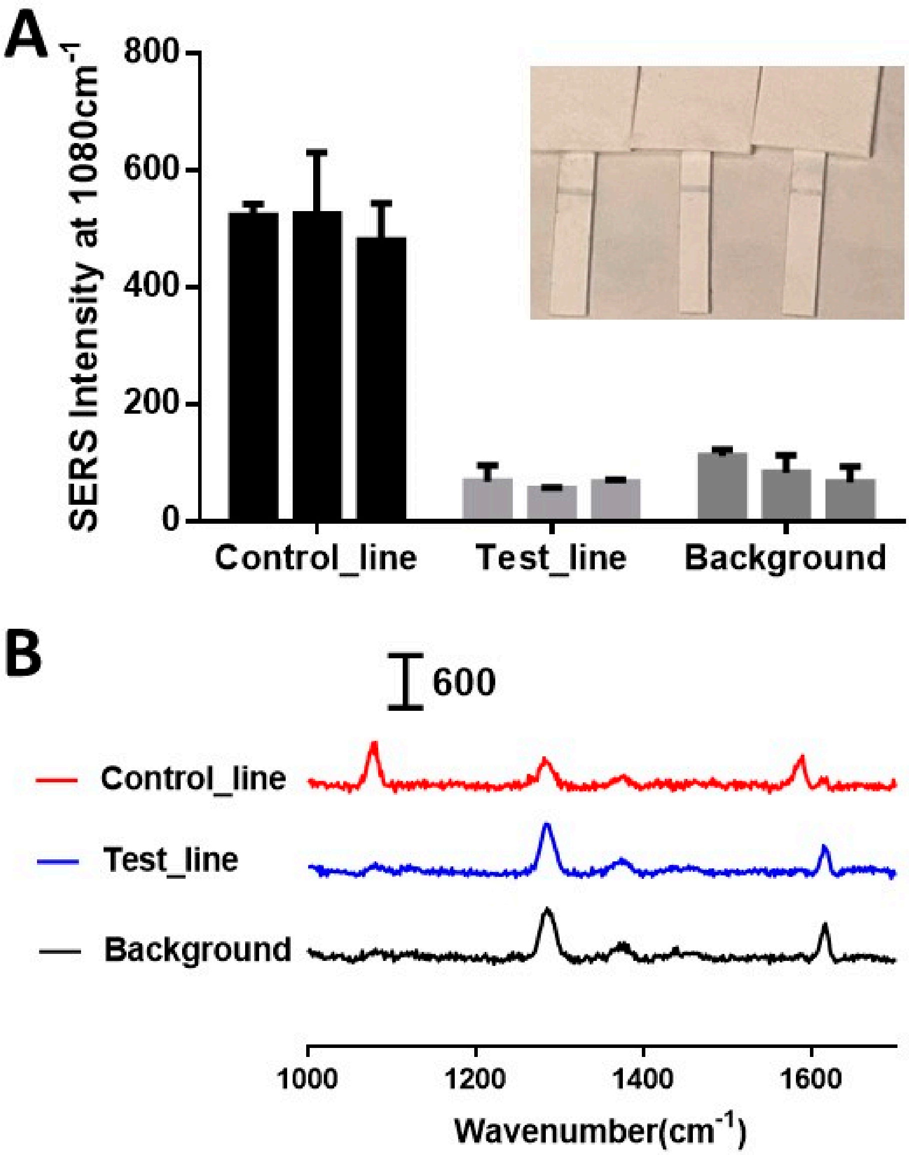

Supplementary Figure S4. Comparative study of non-specific targets, hsa-miR-20a-5p (2µL of 15pg/µL hsa-miR-20a-5p is spiked in 198µL of the serum sample) ; A: SERS Intensity at 1080cm^{-1} and pictures of the LFA assay for hsa-miR-20a-5p, respectively.; B: Representative SERS spectra captured at control line, test line and background of the LFA assay for hsa-miR-20a-5p respectively.
